# Support Vector Machines, Multidimensional Scaling and Magnetic Resonance Imaging Reveal Structural Brain Abnormalities Associated With the Interaction Between Autism Spectrum Disorder and Sex

**DOI:** 10.3389/fncom.2018.00093

**Published:** 2018-11-26

**Authors:** Andrei Irimia, Xiaoyu Lei, Carinna M. Torgerson, Zachary J. Jacokes, Sumiko Abe, John D. Van Horn

**Affiliations:** ^1^Laboratory of Neuro Imaging, Keck School of Medicine, USC Mark and Mary Stevens Neuroimaging and Informatics Institute, University of Southern California, Los Angeles, CA, United States; ^2^Ethel Percy Andrus Gerontology Center, Leonard Davis School of Gerontology, University of Southern California, Los Angeles, CA, United States

**Keywords:** autism, machine learning, support vector machine, neuroimaging, MRI, DTI

## Abstract

Despite substantial efforts, it remains difficult to identify reliable neuroanatomic biomarkers of autism spectrum disorder (ASD) based on magnetic resonance imaging (MRI) and diffusion tensor imaging (DTI). Studies which use standard statistical methods to approach this task have been hampered by numerous challenges, many of which are innate to the mathematical formulation and assumptions of general linear models (GLM). Although the potential of alternative approaches such as machine learning (ML) to identify robust neuroanatomic correlates of psychiatric disease has long been acknowledged, few studies have attempted to evaluate the abilities of ML to identify structural brain abnormalities associated with ASD. Here we use a sample of 110 ASD patients and 83 typically developing (TD) volunteers (95 females) to assess the suitability of support vector machines (SVMs, a robust type of ML) as an alternative to standard statistical inference for identifying structural brain features which can reliably distinguish ASD patients from TD subjects of either sex, thereby facilitating the study of the interaction between ASD diagnosis and sex. We find that SVMs can perform these tasks with high accuracy and that the neuroanatomic correlates of ASD identified using SVMs overlap substantially with those found using conventional statistical methods. Our results confirm and establish SVMs as powerful ML tools for the study of ASD-related structural brain abnormalities. Additionally, they provide novel insights into the volumetric, morphometric, and connectomic correlates of this epidemiologically significant disorder.

## Introduction

Autism spectrum disorder (ASD) is a common (1 in 68 children), strongly-genetic neurodevelopmental disorder which is defined by social communication deficits as well as by the presence of repetitive behaviors and thoughts. ASD is four to five times more prevalent in males (Bloss and Courchesne, 2007; Schumann et al., 2009; Lai et al., 2013; Schaer et al., 2015). Though it is generally acknowledged that structural brain differences exist between ASD patients and typically developing (TD) individuals, the specific nature of these differences has been the subject of intense investigation for at least 20 years (Carper and Courchesne, 2005; Mcalonan et al., 2005). Many brain areas exhibit ASD-related abnormal development; it currently seems unlikely that the behavioral abnormalities associated with this syndrome are due to the typical development of a single brain region (Mcalonan et al., 2005). Instead, studies suggest that spatially-distinct neuroanatomic structures distributed across the entire brain and involved in social information processing tasks may exhibit both structural and functional abnormalities in ASD (Fletcher et al., 1995; Calarge et al., 2003; Schultz et al., 2003). This set of “social brain” regions includes the prefrontal cortex, the medial and ventral portions of the temporal lobe, amygdala, and the cerebellum. Though these and other brain structures are often highlighted as featuring neuroanatomic abnormalities in ASD, the heterogeneity of this patient population greatly complicate the task of evaluating the manner and extent to which this condition affects brain structure.

Despite substantial progress in the use of neuroimaging to characterize and to quantify brain abnormalities in ASD, it remains challenging to establish structural biomarkers of ASD whose sensitivity and specificity are sufficiently high for early clinical diagnosis. This is partly due to methodological limitations which have contributed to contradictory findings across studies and to frequent replication failures. Such limitations include (A) the appreciable heterogeneity of the ASD phenotype (Schaer et al., 2015), (B) the challenges associated with formulating a precise and universally-accepted operational definition of ASD (Volkmar et al., 2009), and (C) many studies' lack of adequate statistical power to characterize structural brain differences between ASD and TD (Bloss and Courchesne, 2007; Schumann et al., 2009; Lai et al., 2013; Schaer et al., 2015).

Many neuroimaging studies of brain structure in ASD have been confronted with limitations which stem from the use of inadequate sample sizes to make statistical inferences. The multiple comparisons problem, for example, involves the increase in the probability of making statistical errors of type I or II as the number of statistical hypotheses being tested on a given sample increases. When making scientific inferences, this problem can be exacerbated if the population being studied exhibits substantial and poorly understood heterogeneity, as in the case of ASD. For reasons such as these, the use of standard statistical approaches—including the general linear model (GLM)—can pose substantial difficulties in ASD neuroimaging research. Such difficulties can manifest themselves in ways which make GLMs particularly unattractive; for example, it is well known that a GLM with more predictor variables than sampling units is underdetermined, which is to say that there are an insufficient number of degrees of freedom (d. f.) to solve its underlying system of linear equations. Because modern neuroimage analysis techniques can provide hundreds and even thousands of quantitative neuroanatomic descriptors, ASD studies may require very large sample sizes if the role of *all* such descriptors in ASD is to be examined. Because there is often substantial co-linearity between many of the neuroanatomic metrics (cortical thickness, volume, area, etc.) which can be computed from magnetic resonance and diffusion tensor imaging (MRI and DTI, respectively) data, there is potential co-linearity between many such variables, leading to the violation of GLM assumptions. Additionally, the task of alleviating this problem by identifying a subset of statistically independent neuroimaging descriptors can involve a prohibitively large number of statistical tests to ascertain their independence, which is again problematic due to the multiple comparisons problem, to its associated high rate of type I and II errors and to the subsequent loss of statistical power. To escape this vicious circle, novel analytic, and inferential approaches may be required.

It was only in the past decade that relatively large neuroimaging datasets became available to the ASD research community (Pelphrey et al., 2011; Di Martino et al., 2014). Notably, recent studies based on the Autism Brain Imaging Data Exchange (ABIDE) repository (http://fcon_1000.projects.nitrc.org/indi/abide) have substantially advanced our understanding of the interaction between ASD and sex (Riddle et al., 2017; Traut et al., 2018; Zhang et al., 2018). Nevertheless, even with the advent of the ABIDE dataset and of the National Database for Autism Research (NDAR, http://ndar.nih.gov), concerns remain that the heterogeneity of ASD may require neuroscientists and clinical researchers to utilize novel and/or alternative analysis methods for understanding the relationship between ASD and its associated structural brain abnormalities.

Machine learning (ML) has achieved considerable prominence as a powerful approach to the identification of robust, early biomarkers of neuropsychiatric disease (Sun et al., 2009; Dyrba et al., 2013). Nevertheless, ML approaches to pattern recognition and classification may not be as intuitive and transparent as those of standard statistical approaches (Duch et al., 2004). Partly for this reason, ML should be evaluated, validated, and compared to standard statistical methods so as to accept it as a valid framework for neuroimaging-based, inferential studies of ASD brain structure abnormalities. In this study, MRI and DTI volumes acquired from a relatively large sample of 110 ASD patients and 83 TD volunteers (95 females) are used to assess the suitability of support vector (SV) machines (SVMs, one of the most popular types of ML) as an alternative to classical inferential statistics for identifying structural brain features which can reliably distinguish ASD patients from TD subjects. More specifically, our purpose is to evaluate the ability of SVMs to distinguish the two samples based on widely-used neuroanatomic descriptors, including the thickness, area, curvature, and volume of gray matter (GM) regions, as well as white matter (WM) connectivity density (CD). To compare and validate our findings against those of standard inferential methods, we implement a *post hoc* statistical analysis to confirm that the structural brain features used for the SVM differ significantly— in a statistical sense—between ASD and TD subjects, and additionally within and between these groups according to sex. Our results confirm and establish SVMs as powerful ML tools for the study of structural brain abnormalities related to ASD; additionally, they provide novel insights into the structural correlates of this clinically significant disorder.

## Materials and methods

### Participants

This study was carried out as part of the Autism Center of Excellence (ACE) Program funded by the National Institute of Mental Health (NIMH). Psychometric and neuroimaging data were acquired at four sites: (1) the Center for Translational Developmental Neuroscience, Yale University, New Haven, CT (73 volunteers); (2) the Laboratory of Cognitive Neuroscience, Boston Children's Hospital, Harvard Medical School, Boston, MA (49 volunteers); (3) the Center on Human Development and Disability, Seattle Children's Hospital, University of Washington School of Medicine, Seattle, WA (92 volunteers); (4) the Staglin IMHRO Center for Cognitive Neuroscience, David Geffen School of Medicine, University of California, Los Angeles, CA (73 volunteers). All research was performed in accordance with US federal law (45 C.F.R. 46) and with the approval of the Institutional Review Boards (IRBs) of both the University of Southern California (USC) and the four institutions where the neuroimaging and psychometric data had been acquired. Recruited subjects included *N*_1_ = 110 ASD patients (55 males) and *N*_2_ = 83 TD subjects (43 males), for a total of *N* = *N*_1_ + *N*_2_ = 193 volunteers. All subjects and their legally authorized representatives provided informed written consent. The Differential Ability Scales (DAS-II)—including the Verbal (V), Non-Verbal (NV), Spatial (S), General Conceptual Ability (GCA), and Spatial Non-Verbal Composite (SNC) scales—were used to assess the cognitive abilities of the participants. SNC scores were unavailable for 9 subjects and one additional subject did not have DAS scores available. Welch's *t*-test for samples with unequal variances was used to evaluate the significance of differences in age and DAS scores between the two cohorts. To test the statistical significance of the difference in sex composition between the two groups, sex was coded as a binary variable and a χ^2^ test was used.

### Inclusion criteria

ASD patients were included based upon the results of an evaluation by an experienced clinician, together with the Autism Diagnostic Observation Schedule (ADOS-2) and of the Autism Diagnostic Interview (ADI). The ADOS inclusion criterion was a comparison score above 3; the ADI inclusion criteria were: (*i*) a communication total (R) score above 8; (*ii*) a behavioral (S) total score above 6; (*iii*) a social affect (T) total score above 1; (*iv*) a sum of the previous three above 18, i.e., R + S + T > 18. A volunteer had to satisfy both ADI and ADOS criteria and had to meet clinical (DSM-5) criteria to qualify for inclusion.

### Exclusion criteria

For the TD group, exclusion criteria included suspected, referred or diagnosed ASD, learning/intellectual disability, schizophrenia, the presence of any other psychiatric or developmental disorders, as well as the existence of a first- or second-degree relative who had ASD. Exclusion criteria for the ASD group included any psychiatric, neurological or genetic comorbidity (including—but not limited to—the use of any barbiturate, benzodiazepine or anti-epileptic medication, pregnancy, active tic disorders, fragile X syndrome, spasms, epilepsy, pre-/peri-natal birth injury, brain damage, severe psychological or nutritional deprivation, auditory or visual impairment after correction, as well as sensorimotor difficulties which precluded the valid use of diagnostic instruments).

### Recruitment protocol

Research-reliable clinicians screened potential enrollees either in person or by telephone to ensure that all inclusion and exclusion criteria were satisfied. Prior to enrollee visits, phone interviews with parents were carried out. During in-person visits, examiners with research levels of ADOS and/or ADI reliability collected medical history information and also acquired ADOS and ADI measures by direct observation.

### MRI acquisition

MRI data were collected using Siemens scanners (TrioTim or Prisma^fit^) with magnetic field strengths of 3 T. Subject head movements in the MR scanner coil were restricted through ample padding as well as by using headphones and video goggles. A magnetization-prepared rapid acquisition gradient echo (MP-RAGE) sequence was used to acquire *T*_1_-weighted volumes; the parameters of the sequence were: 256 interleaved, single-shot, sagittally-oriented, slices; 256 mm field of view (FOV); 1 mm slice thickness; 256 × 256 acquisition matrix; a repetition time (*T*_*R*_) of 2,530 ms; an echo time (*T*_*E*_) of 3.31 ms (TrioTim scanners) or 3.34 ms (Prisma scanners); an inversion time (*T*_*I*_) of 1,100 ms; a flip angle of 7-degrees; phase and slice resolutions of 100%; a bandwidth of 200 Hz/pixel (Px) and an echo spacing of 7.6 ms. To acquire 64-direction DTI volumes, the following acquisition parameters were used: 96 × 96 acquisition matrix; 190 mm FOV; 60 transversally-oriented, interleaved slices with a thickness of 2 mm; a *T*_*R*_ of 9,000 ms (TrioTim scanners) or 7,300 ms (Prisma scanners); a *T*_*E*_ of 93 ms (TrioTim scanners) or 74 ms (Prisma scanners); a flip angle of 90 degrees; a phase resolution of 100%; B_0_ values of 0 s/mm^2^ and 1,000 s/mm^2^; a bandwidth of 2,264 Hz/Px bandwidth (TrioTim scanners) or 1,680 Hz/Px (Prisma scanners); and an echo spacing of 0.69 ms. All the neuroimaging data were de-identified, encrypted, and transferred to the Data Coordinating Center (DCC) residing at the Laboratory of Neuro Imaging (LONI) in the USC Mark and Mary Stevens Neuroimaging and Informatics Institute. Quality control (QC) and protocol compliance were undertaken via the LONI QC System (http://qc.loni.usc.edu) and data were also stored in the NDAR database (http://ndar.nih.gov). Instances of head motion during the scan were noted by site investigators; whenever this appeared to degrade image quality appreciably, the subjects in question were excluded from an original dataset prior to compilation and detailed quantitative analysis of the dataset used in this study.

### Image processing

Image processing was performed using the LONI Pipeline environment (http://pipeline.loni.usc.edu). Detailed descriptions and visual representations of the workflow and processing environment are available elsewhere (Dinov et al., 2009, 2010). For each subject, affine co-registration of DTI and MRI volumes was performed. Subsequently, each DTI volume was corrected for eddy currents and then processed using TrackVis (http://trackvis.org) to recreate WM fiber streamlines via deterministic tractography. If a streamline exhibited a turning angle below 60 degrees, it was discarded. A triangular tessellation with ~300,000 vertices (average inter-vertex distance: ~1 mm) was used to reconstruct the cortical surface of the brain and to produce an anatomically-faithful, smooth representation of the GM/ WM interface (Fischl et al., 1999). At each tessellation vertex *v*_*i*_, FreeSurfer 6.0 software was used to measure the cortical thickness as the distance between the GM/WM boundary and the cortical surface. As described elsewhere (Destrieux et al., 2010), a probabilistic atlas was used to identify and parcel a total of 74 cortical structures (gyri and sulci) in each hemisphere of the brain. After also segmenting the brain stem, this resulted in a total of 165 segmented parcels over the entire brain. Probabilistic information estimated from a manually labeled training set was used to assign neuroanatomical labels to voxels using a method whose accuracy is comparable to that of manual labeling (Fischl et al., 2004). After the parcellation of each structure, its surface area, volume, and mean curvature were calculated. To avoid the confounding effect of head size, every measure was also normalized by total intracranial volume (TICV) prior to further analysis.

### Connectivity calculation

Cortical inter-connectivity values were calculated as detailed elsewhere (Irimia et al., 2012). Briefly, the mean fractional anisotropy (FA) of each WM streamline bundle was calculated as the average FA over all DTI voxels traversed by the fiber bundle along its path. Mean FA maps for connections innervating each point on the cortex were smoothed across using a circularly symmetric Gaussian kernel with a full width at half maximum of 5 mm and averaged across subjects using a non-rigid, high-dimensional spherical averaging method to align cortical folding patterns (Fischl et al., 1999). After cortical parcellation and streamline tractography, the WM connectivity matrix of each subject was calculated as follows. Let *v*_*i*_ and *v*_*j*_ be cortical mesh vertices linked by some WM connection *c*_*ij*_. For each such connection, the three-dimensional coordinates associated with the extremities of *c*_*ij*_ (i.e., with *v*_*i*_ and *v*_*j*_) were identified. The corresponding entry indexed by *i* and *j* in the connectivity matrix **C** of each subject was assigned an appropriate value to reflect the presence of a connection between *v*_*i*_ and *v*_*j*_. This process was repeated for each connection. The mean FA of *c*_*ij*_ was computed as the average of FA values over all DTI voxels traversed by *c*_*ij*_ from one end of each connection to the other end. Similarly, at each vertex *v*_*i*_ on the cortical mesh, the mean FA of connections linking *v*_*i*_ to the rest of the brain was computed. The CD at each vertex was calculated as the sum of all streamlines linking it to the rest of the brain, divided by the area of the vertex neighborhood in question and by the total number of brain connections. Here, the neighborhood of vertex *v*_*i*_ denotes the portion of the mesh surface containing points which are closest to *v*_*i*_, i.e., points whose geodesic distance from *v*_*i*_ does not exceed 5 mm.

### Statistical feature extraction

**Figure 1:**
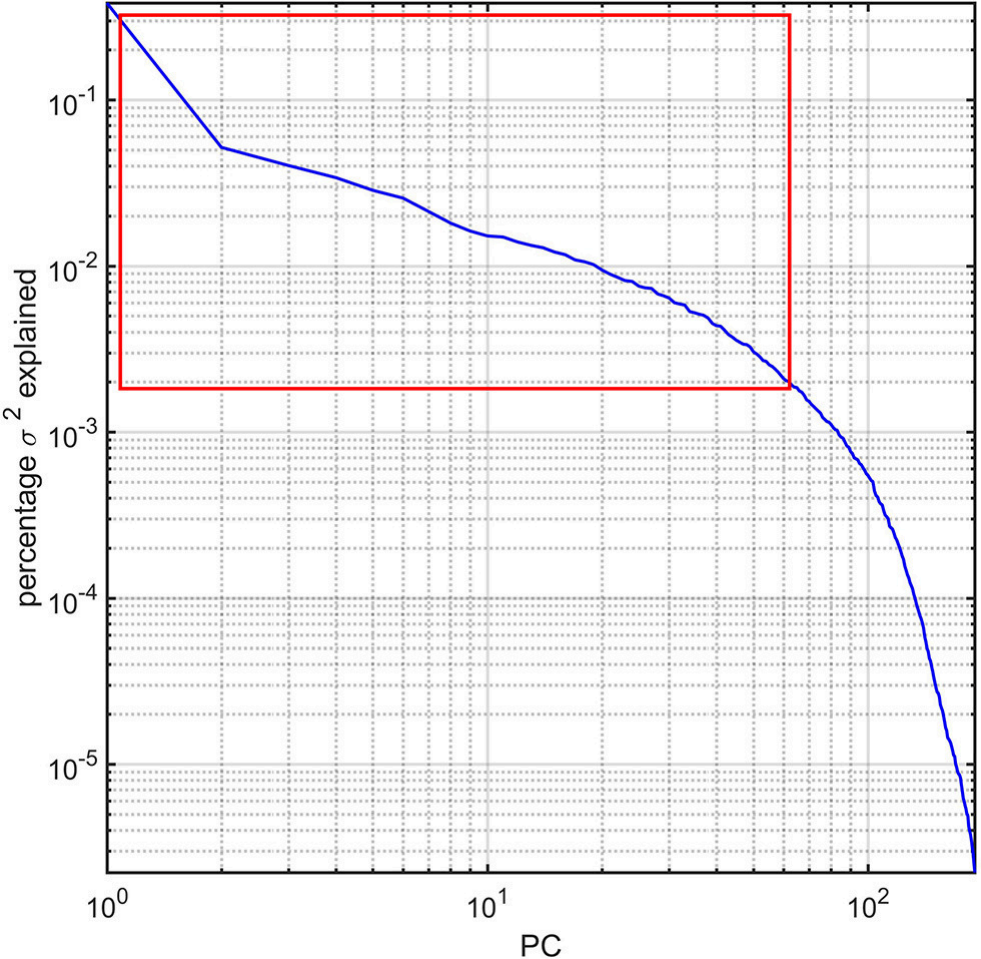
Percentage variance (σ^2^) explained by the PCs of the study dataset. The first ~63 PCs were found to explain ~95% of the total variance and are highlighted by a red rectangle. All PCs are sorted in descending order of the percentage σ^2^ explained. The log-log plot obviates how components beyond the ~63rd PC contribute to σ^2^ negligibly.

For each subject in the study, the image processing steps detailed previously provide us with values for the GM thickness, volume, cortical area, mean curvature, and CD of 165 brain regions, amounting to a total of 825 metrics available for 193 subjects. The dimensionality of this dataset was reduced via principal component analysis (PCA) by first sorting the eigenvalues of the covariance matrix in descending order according to the percentage of the variance in the data which they explained. The number of PCs selected for subsequent analysis was selected so that the sum of their percentage of variance explained exceeded 95%. This threshold was selected after (A) examining the plot of the percentage of variance explained by each eigenvalue and then (B) identifying the sharp cutoff point beyond which additional eigenvalues contributed to the total variance explained by a relatively negligible amount (Figure 1). This approach to dimensionality reduction is standard in a variety of scientific applications (Rencher, 2002). The MATLAB function *princomp* was used to implement the PCA.

### SVM classification

Subsets of each population sample were used to train an SVM classifier to distinguish (A) between ASD and TD and (B) between ASD males, ASD females, TD males, and TD females based on their brain features as selected using PCA. Three separate analyses were implemented. In the first of these, the ability of the SVM to separate the groups using *both* structural (area, volume, thickness, curvature) and connectomic (CD) variables was investigated. In the second analysis, only structural features (area, volume, thickness, curvature) were used; in the third analysis, only connectomic (CD) features were used. In all analyses, before training, data points were automatically centered at their mean and then scaled to have unit standard deviation. To take into account the unbalanced group design, box constraint values of *N*/(2*N*_1_) and *N*/(2*N*_2_) were used for the TD group and ASD group, respectively. When additionally investigating within- and between-group differences predicated on sex, box constraint values were adjusted accordingly. All the variables were constrained to satisfy the Karush-Kuhn-Tucker (KKT) conditions for training (Cristianini and Shawe-Taylor, 2000), which use the auxiliary Lagrangian function

(1) $$\begin{array}{r} L\left(x,\lambda \right)=f\left(x\right)+\sum_{i}\lambda_{g,i}g_{i}\left(x\right)+\sum_{i}\lambda_{h,i}h_{i}\left(x\right) \end{array}$$

Above, *f*(*x*) is the objective function of the classifier whereas *g*(*x*) and *h*(*x*) are vectors of constraint functions subject to *g*(*x*) ≤ 0, and *h*(*x*) = 0. The vector λ = [λ_*g*_ λ_*h*_] is the Lagrange multiplier vector. Additional KKT conditions are: ∇_*x*_*L*(*x*, λ) = 0, λ_*g, i*_*g*_*i*_(*x*) = 0 and λ_*g, i*_ ≥0. These constraints are analogous to the condition that the gradient must be zero at a minimum subject to the constraints. Tolerance values of 0.001 (for two-group classification) and 2^−0.77^ ≈0.58 (for four-group classification) were used for checking that these were satisfied. The latter value was obtained by identifying a suitable value of the optimization parameter *k* such that a tolerance threshold of 2^*k*^ could yield an optimal solution to the optimization problem of separating hyperplanes using sequential minimal optimization (SMO). For two-group classification, a scaling factor of 1 was used in the radial basis function kernel and a linear kernel function was used to map the training data into kernel space. For four-group classification, a second-degree polynomial kernel and a one-vs.-one (OVO) function were used for space mapping. After this step, a binary classifier was built to distinguish between each pair of classes from the original training set; this resulted in six binary classifiers for four-group classification. The full classifier was used to assign a class membership to each subject based on the highest vote among all binary classifiers. A penalty parameter with a value of 2^−16^ was used to specify the threshold for misclassification during four-group classification and a grid search for an optimal solution to the optimization problem was implemented with γ = 0.25, where γ specifies the threshold for the variance of the corresponding Gaussian distributions around support vectors. For all classifiers, computed quantities included the SVs, their weights, the intercept of the hyperplane separating the groups in normalized data space and the kernel function. In the following stage, the data *x* were classified using the trained SVM classifier according to the following equation:

(2) $$\begin{array}{r} c=\sum_{i}a_{i}k\left(s_{i},x\right)+b, \end{array}$$

where *s*_*i*_ are the SVs, *a*_*i*_ are the weights, *k*(._,_.) is the kernel function and *b* is the bias. If *c*≥0, then *x* is classified as member of the first group, otherwise it is classified as a member of the second group. The SVM algorithm was implemented in MATLAB for this specific study. Disjoint, random data partitions for 10-fold cross-validation were created and the classifier was cross-validated using these partitions. The SVM classification process was repeated 500 times and the descriptive statistics (mean, standard deviation) of the classification accuracy were calculated.

### A posteriori statistical analysis

To confirm the ability of the SVM to separate the two cohorts, an *a posteriori* statistical analysis was implemented. First, age- and site-related effects were regressed out and Welch's two-sample *t*-test for samples with unequal variances was subsequently used to test the null hypothesis that the mean of each feature variable did not differ significantly between the ASD group and the TD group. In the case of four-group classification, after regressing out age- and site-related effects, an analysis of variance (ANOVA) for samples with unequal variances was used to test the null hypothesis that the mean of each feature variable did not differ significantly among ASD males, ASD females, TD males, and TD females. Hypotheses were rejected at a significance level α ≤ 0.05. Corrections for multiple comparisons were implemented using the false discovery rate (FDR) approach of Benjamini and Hochberg (1995).

### Multidimensional scaling and visualization

To understand and visualize how near or far subjects are from each other in the multidimensional space defined by their structural brain feature variables, multidimensional scaling (MDS) was undertaken using the Euclidian distance as its dissimilarity measure. The data configuration matrix **Y** was computed, as were the eigenvalues of **YY**^*T*^. The corresponding eigenvectors were arranged in descending order of their explained variance, and data points were projected along the first three eigenvectors to generate a three-dimensional (3D) representation which conveyed the extent to which the SVM was successful in separating the two cohorts based on their structural brain variables. The INVIZIAN visualization environment (Bowman et al., 2012) was used to represent the cortical surface of each subject at 3D coordinate locations specified by the MDS eigenvectors.

## Results

Participant demographics and their scores are summarized in Tables 1 and 2. No statistically significant difference between the ASD group and the TD group was found either in age (*t*_191_ = −0.86, *p* > 0.39) or DAS scores (DAS-V: *t*_191_ = 1.07, *p* > 0.28; DAS-NV: *t*_191_ = 0.72, *p* > 0.48; DAS-S: *t*_191_ = −0.44, *p* > 0.66; DAS-GCA: *t*_191_ = 0.92, *p* > 0.36; DAS-SRC: *t*_191_ = 0.51, *p* > 0.61). Similarly, no significant difference in either the sex composition of the two samples (χ^2^ = 0.13, d. f. = 1, *p* > 0.72) or in their TICVs (Welch's *t* = 1.14, d. f. = 187.54, *p* > 0.13) was found.

**Table 1:** ASD and HC cohort demographics and DAS scores by domain.

			**DAS**				
**Cohort**	**Age [years]**	**Sex ratio**	**V**	**NV**	**S**	**GCA**	**SNC**
ASD	12.74 (2.79)	1.01:1	102.56 (20.23)	101.17 (17.81)	99.79 (17.17)	101.65 (19.44)	100.78 (18.15)
HC	13.04 (2.95)	1.08:1	109.95 (15.64)	107.13 (14.82)	105.13 (13.26)	108.86 (14.78)	107.20 (13.73)

*V, verbal; NV, non-verbal; S, spatial; GCA, general conceptual ability; SNC, spatial non-verbal composite*.

*The sex ratio is reported as the number of males for every female in each sample. Averages are reported, and the standard deviation is shown in parentheses*.

**Table 2:** ASD volunteers' mean scores for each ADOS domain.

**ADOS**				**ADI**		
**AB**	**AD**	**AB + AD**	**C**	**T**	**R**	**S**
9.30 (3.57)	2.58 (1.76)	11.88 (4.25)	6.78 (2.04)	19.13 (5.41)	16.06 (4.25)	5.90 (2.45)

*AB, social affect; AD, behavior; AB + AD, overall total score; C, comparison score and for each ADI domain T, social affect; R, communication; S, behavior, with standard deviations in parentheses*.

Because the SVM classification process was implemented 500 times, the results reported below pertain to the most frequent classification scenario; furthermore, the means and standard deviations reported below for various results and metrics were also calculated over all 500 scenarios. The mean and standard deviation of the PC number obtained upon dimensionality reduction and then used for SVM input was 63.21 ± 1.43. In the first analysis, in the classification scenario encountered most frequently among the 500 considered, we identified an average of 11.12 ± 0.71 brain structures (see Table 3) whose associated neuroanatomic descriptors allowed the subjects in the two cohorts to be classified with an accuracy of 93.26% based on 10-fold cross-validation. The standard deviation of this two-group classification accuracy over 500 scenarios was 4.21%. Moreover, the same descriptors allowed the subjects in the four cohorts to be classified with an accuracy of 94.82% based on 10-fold cross-validation; the standard deviation of this four-group classification accuracy over 500 scenarios was 5.56%. In the second and third analyses, TD and ASD subjects—regardless of sex—could be classified with accuracies of 74.09 ± 7.52% and 55.96 ± 9.15%, respectively; by contrast, the four cohorts of male TD, female TD, male ASD, and female ASD subjects could be classified with accuracies of 61.14% ± 10.75% and 49.22% ± 12.34%, respectively. These results clearly indicate that the accuracy of the SVM classification was superior when structural and connectomic features were used together. For this reason, only the results of the first analysis are discussed in what follows and all subsequently-reported results should be understood to be associated with the first analysis.

**Table 3:** SVM-identified brain structure measures which can together distinguish among patients based on their diagnosis (i.e., ASD vs. TD, Welch's *T* test) or on the interaction between sex and diagnosis (two-way ANOVA).

			**Diagnosis**			**Diagnosis** × **Sex**	
**Anatomic structure**	**Hemisphere**	**Measure**	***t***	**d. f**.	***p***	***F*_(3, 189)_**	***p***
**FRONTAL LOBE**							
Medial orbital sulcus	Right	Thickness	2.388	189.649	0.018*	3.113	0.027*
Straight gyrus	Right	CD	2.124	187.787	0.035*	2.351	0.074*
	Left	Thickness	2.150	183.505	0.033*	3.409	0.019*
	Left	Volume	2.190	186.758	0.030*	4.618	0.004*
Inferior frontal gyrus, orbital part	Left	CD	−2.138	170.856	0.034*	2.655	0.050*
**TEMPORAL LOBE**							
Temporal pole	Left	Curvature	2.909	190.090	0.004*	3.929	0.009*
	Right	Thickness	−1.971	183.857	0.049*	2.883	0.037*
Parahippocampal gyrus	Right	Volume	−2.026	166.292	0.044*	3.394	0.019*
	Left	Volume	−2.304	190.792	0.022*	2.254	0.083*
Superior temporal gyrus	Right	Curvature	2.080	182.553	0.039*	3.052	0.030*
**LIMBIC LOBE**							
Isthmus of the cingulate gyrus	Right	Volume	2.276	188.168	0.024*	3.606	0.014*
	Left	Area	2.543	186.679	0.012*	3.002	0.032*
Pericallosal sulcus	Left	Area	2.318	178.371	0.022*	2.320	0.077*
	Right	Area	1.962	189.338	0.049*	1.271	0.285*
**OCCIPITAL LOBE**							
Cuneus	Right	Area	2.223	186.596	0.027*	3.676	0.013*
	Left	Area	1.957	185.521	0.049*	1.976	0.119*
Superior and transverse occipital sulci	Left	CD	2.069	181.599	0.040*	1.837	0.142*
Occipital pole	Right	Area	2.454	190.459	0.015*	4.570	0.004*
	Left	Area	2.443	180.449	0.016*	3.536	0.016*

*In the first case (diagnosis), a posteriori statistical analysis indicates that all identified measures exhibit statistically significant differences between the study (ASD) group and the control (TD) group. Welch's T-test with a significance level of α < 0.05 was used; t statistics, the associated d. f. and p-values are reported. A positive t statistic indicates that a corresponding measure's mean over the study (ASD) group is significantly larger than its mean over the control (TD) group. A negative t statistic indicates the reverse. To estimate the d. f., Welch's T-test relies on the Welch-Satterthwaite approximation, which involves the variances of the two samples. Thus, unlike in the case of Student's T-test, the d. f. of Welch's T test can differ whenever sample variances also differ. In the second case (diagnosis × sex interaction), a two-way ANOVA (factors: ASD diagnosis, sex) was used to identify the brain features which, in addition to being able to distinguish ASD from TD subjects, can also distinguish these subjects based on their sex. In this second analysis, the F statistic has 3 and 189 d. f. In both analyses, the null hypothesis is rejected at a significance level α < 0.05 subject to a multiple comparison correction, and statistically significant p-values are marked with an asterisk*.

Confusion matrix elements are reported below for the most frequent classification scenario. In what follows, a positive (P) refers to a subject being classified as having ASD; a negative (N) refers to a subject being classified as TD. When attempting to distinguish between ASD and TD subjects using *both* structural and connectomic features, the number of true positives (TP, i.e., ASD subjects correctly classified as such) was found to be 103 (53.37%) and the number of true negatives (TN, i.e., TD subjects correctly classified as such) was found to be 77 (39.90%). The number of false negatives (FN, i.e., TD subjects incorrectly classified as ASD subjects) was 7 (3.62%) and the number of false positives (FP, i.e., TD subjects incorrectly classified as ASD subjects) was 3 (1.55%). These results translate into a sensitivity of 97.17% and a specificity of 91.67%.

When attempting to distinguish between ASD males (P_♂_), ASD females (P_♀_), TD males (N_♂_), and TD females (N_♀_) at once, the confusion matrix is replaced by a confusion tensor of third rank, whose entries were found to be TP_♀_ = 52 (26.94%), TN_♂_ = 39 (20.21%), TP_♀_ = 54 (27.98%), TN_♀_ = 38 (19.69%), FP_♂_ = 3 (1.55%), FN_♂_ = 4 (2.07%), FP_♀_ = 1 (0.52%), and FN_♀_ = 2 (1.04%). These results yield a sensitivity of 96.36% and a specificity of 92.77%.

**Figure 2:**
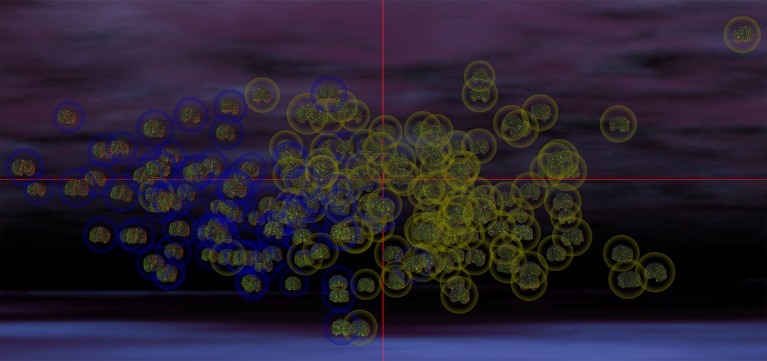
MDS representation which illustrates the ability of structural brain variables to distinguish ASD patients from TD subjects. The cortical surface of each participant is shown, with the brains of participants belonging to each group being surrounded by a circle whose color indicates cohort membership (ASD in yellow, TD in blue). The spatial coordinates of each brain are specified by the MDS projection of each volunteer's brain structure descriptors from a hyperspace containing all descriptive variables to 3D space. Each coordinate axis in this 3D representation corresponds to each of the first three MDS eigenvectors of the matrix **YY**^*T*^ (see Methods), accounting for the largest amount of variance in the data. In this representation, any pair of subjects whose brains are located farther apart from each other differ more in their structural features than pairs of subjects whose brains are closer. The excellent separation between the two cohorts is apparent due to the clustering of ASD patients away from the cluster of TD subjects. The visualization was produced within the INVIZIAN software package (Bowman et al., 2012).

In the most frequent classification scenario, the properties of several structures in the ventral aspect of the frontal lobe were found to differ significantly and bilaterally, with cortical thickness, volume, and CD being implicated in these differences. The properties of the temporal pole, parahippocampal, and superior temporal gyri were also found to differ; in particular, parahippocampal volume was found to be significantly smaller in the ASD group than in the TD group. The areas and volumes of some limbic structure—such as the cingulate gyrus and pericallosal sulcus—were also found to differ, with larger areas and volumes in the ASD group. The cuneus, occipital poles as well as the superior and transverse occipital sulci were found to have larger areas and CDs in the ASD group compared to the TD group. The ability of structural brain variables to discriminate between ASD and TD children is confirmed by the 3D MDS representation in Figure 2, which confirms the excellent separation between groups and captures the ability of the SVM to distinguish ASD from TD based on neuroanatomic descriptors.

**Figure 3:**
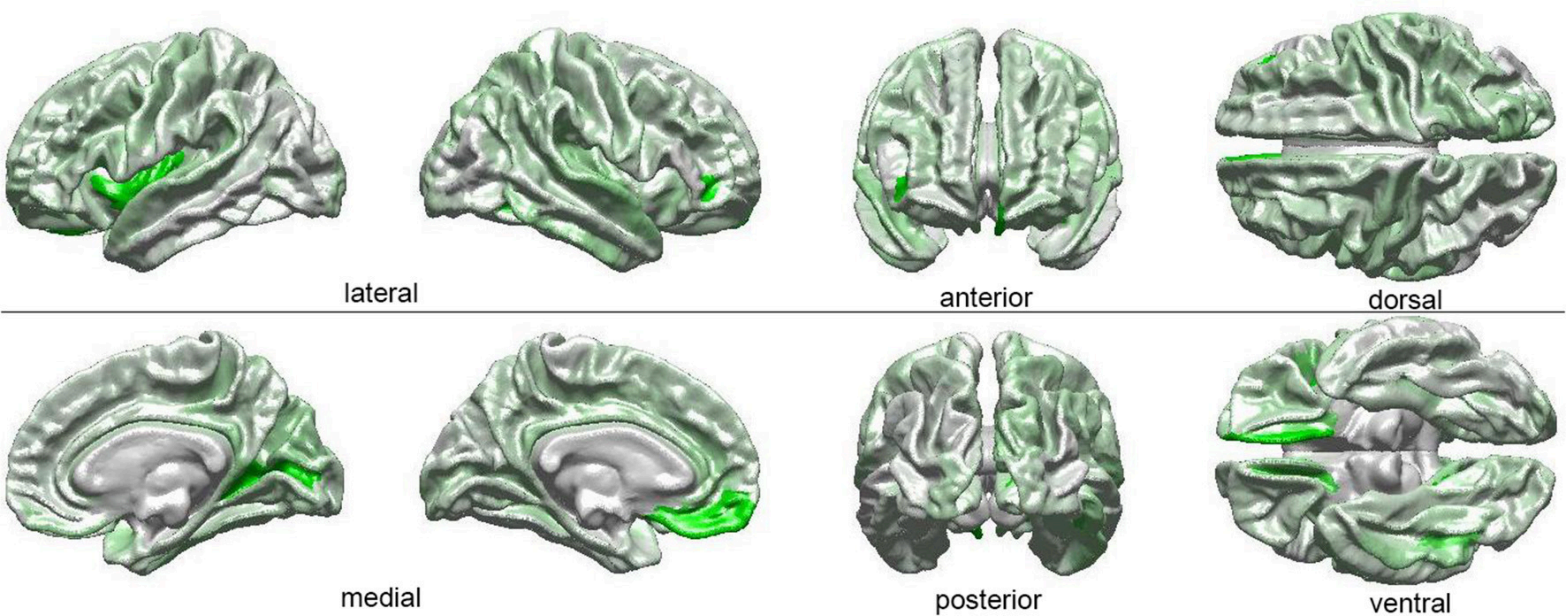
Visual depiction of SVM-identified brain regions whose volumetric, morphometric, and/or connectomic features can together distinguish among patients based on the interaction between sex and diagnosis. First, *a posteriori* two-way ANOVA (factors: ASD diagnosis, sex) revealed brain locations whose features, in addition to being able to distinguish ASD from TD subjects, can also distinguish these subjects based on their sex (see Table 1 and text). Then, the ANOVA test statistic (*F*_3_, _189_) was plotted on the cortical surface of an average brain, with color shades at each cortical location encoding the *F* statistic, which ranges from 0 (white) to a maximum of 4.618 (bright green).

The results of our findings are illustrated in Figure 3 and summarized in Table 3. As both of these indicate, only some of the brain features which can distinguish ASD subjects from TD volunteers play a significant role in predicating the diagnosis-by-sex interaction. For example, the left **temporal pole** of ASD females was found to have higher curvature (Welch's *t* = −2.174, d. f. = 102.026, *p* < 0.032) than in ASD males; no such difference was found to exist between TD females and TD males (Welch's *t* = −0.768, d. f. = 76.873, *p* > 0.445), consistent with previous observations (Hartley and Sikora, 2009). The volume of the right **parahippocampal gyrus** was found to be larger (Welch's *t* = −2.402, d. f. = 102.776, *p* < 0.018) in ASD females compared to ASD males, and the curvature of **superior temporal gyrus** was greater (Welch's *t* = -2.088, d. f. = 104.453, *p* < 0.039) in ASD females than in ASD males, in agreement with previous findings on differential memory processing abilities between sexes in ASD (Baron-Cohen et al., 2005). The surface area of the **occipital poles** (left hemisphere: Welch's *t* = −2.112, d. f. = 101.984, *p* < 0.037; right hemisphere: Welch's *t* = −2.533, d. f. = 99.729, *p* < 0.013) and right **cuneus** (Welch's *t* = −2.235, d. f. = 99.089, *p* < 0.028) were found to be larger in ASD females than in ASD males; no such sex-related differences were found in TD volunteers. When interpreted together, our results appear to indicate that sex-related differences in ASD incidence are associated with brain features which are not only ASD-specific but also sex-specific. Furthermore, only a subset of ASD-related structural brain features are involved in predicating the interaction between ASD diagnosis and sex.

## Discussion

To assess the suitability of SVM classifiers for the study of ASD-related neuroanatomical abnormalities, it is useful to evaluate the extent to which their results are consistent with those of previous studies, and particularly with findings obtained using very different inferential approaches. To this end, in what follows, the SVM findings summarized in Table 3 are discussed in relation to the existing body of literature on the neuroanatomical correlates of ASD.

The present study identifies medial, ventromedial, and dorsolateral regions of the frontal lobe as being associated with ASD-related neuroanatomical abnormalities. The right **medial orbital sulcus**, for example, has been implicated in ASD-related social impairment (Amaral et al., 2008), in repetitive or stereotyped behaviors, in the abnormal actions of obsessive-compulsive disorder (Whiteside et al., 2004; Atmaca et al., 2007), in decision making and in expectation rewarding (Kringelbach, 2005), all of which are affected in ASD. Here, the cortical thickness of this structure is found to be greater in ASD than in TD, which confirms the findings of Carper and Courchesne (2005); in addition, as Table 3 suggests, this brain feature partially mediates the ASD diagnosis-by-sex interaction. The **straight gyrus** (*gyrus rectus*) is involved in attentional control and its functions are tightly integrated with those of orbital cortex (Nestor et al., 2015); we find this structure to exhibit bilateral structural abnormalities in ASD, and the left straight gyrus is found to mediate the statistical interaction of ASD diagnosis and sex. The **inferior frontal gyrus**, which overlaps with Broca's area (Brodmann's areas 44 and 45) is heavily involved in expressive language function, which is negatively impacted by ASD (Redcay, 2008). The shape of this structure was previously found to be abnormal in autism (Levitt et al., 2003; Nordahl et al., 2007); and the findings of the present study indicate that, in the left hemisphere, the CD of this gyrus is significantly lower in ASD than in TD. This parallels, to some extent, the communication-related deficits observed in ASD patients (Redcay, 2008). Because Jiao et al. (2010) reported the inferior frontal gyrus as exhibiting significantly thinner GM in ASD than in TD, our findings and those of Jiao et al. together suggest that ASD-related structural abnormalities in this region may be modulated simultaneously by both WM connectivity and GM structure. This hypothesis is supported by the results of Pardini et al. (2009), who found differences in the mean FA of WM tractography bundles connecting the inferior dorsolateral and orbitofrontal cortices of ASD patients to the rest of the brain. It should be noted that this region was previously identified by Ecker et al. (2010) as being particularly important for distinguishing between ASD and TD volunteers when using SVMs.

Concerning temporal lobe structures, it has been proposed that the **temporal pole** is involved in social cognition (Schultz, 2005) and that it exhibits lower structural connectivity in ASD compared to TD (Roine et al., 2015). Its role in ASD-related resting-state functional connectivity abnormalities is also prominent (Venkataraman et al., 2015), and Table 3 indicates that the cortical thickness of the right temporal pole in greater in TD than in ASD, in agreement with findings from other studies (Boddaert et al., 2004; Jiao et al., 2010). The **parahippocampal gyrus**, which play an important role in contextual fear and in the interactions between emotion and cognition (Ke et al., 2008), is found to be bilaterally and significantly smaller in ASD, which confirms previous findings by other researchers (Ecker et al., 2010; Jiao et al., 2010). The functions of this structure are intimately related to those of the **superior temporal gyrus**, which is implicated in social behavior according to several lines of evidence provided by animal studies, human lesion research, and by functional imaging (Adolphs, 2001). The present study indicates that the curvature of this structure is abnormal in ASD, a conclusion which is supported by previous studies (Levitt et al., 2003; Nordahl et al., 2007). It is important to note that our findings appear to be congruent with those reported recently by Ecker et al. (2017); these researchers found spatial clusters of significant sex-by-diagnosis interaction when comparing the cortical thickness of ASD subjects to that of HC individuals. Despite confounds—e.g., children in our study vs. adults in Ecker et al.'s—it is interesting that both studies found the sex-by-diagnosis interaction to be mediated by structural brain differences localized in the ventral and medial aspects of the temporal lobe (e.g., temporal pole, parahippocampal area, superior temporal gyrus). These are regions implicated in the ventral processing stream of attention, working memory, and salience (Hickok and Poeppel, 2004), on whose developmental stages little is known even though the cognitive processes associated with this stream are frequently affected in ASD (Gathers et al., 2004). Whereas the dorsal stream appears to experience problematic developmental trajectories in ASD (Spencer et al., 2000), it is not well understood whether these trajectories are modulated by the interaction of ASD diagnosis and sex. Future studies should test the hypothesis that, in ASD, the structural features of brain areas recruited by the ventral processing stream may reflect functional abnormalities modulated by the interaction of ASD diagnosis with sex.

Pertaining to the limbic lobe, the isthmus of the right **cingulate gyrus** is found to have a larger volume in ASD, which confirms the findings of Jiao et al. (2010). This structure connects the limbic lobe to the parahippocampal gyrus and plays an important role in the encoding of memories associated with executive function and cognitive control, both of which are affected by ASD (Hadland et al., 2003). The **pericallosal sulcus**, which is anatomically adjacent to the cingulate gyrus and functionally related to it, is found here to have a larger area in ASD patients compared to TD subjects. This is perhaps unsurprising given the abundance of evidence to the effect that the structural properties of the corpus callosum are substantially different in ASD compared to TD (Hardan et al., 2000; Barnea-Goraly et al., 2004; Herbert et al., 2004; Alexander et al., 2007; Just et al., 2007). A neuroimaging study meta-analysis undertaken by Stanfield et al. (2008) identified substantial consensus in the ASD literature to the effect that the volume of the corpus callosum is smaller in ASD compared to TD (Pua et al., 2017). A relatively thin corpus callosum implies that the distance between it and the cingulate gyrus is longer; however, since the grove between the two structures is the pericallosal sulcus, our finding is consistent with the notion that the latter region has a larger area in ASD.

It is widely acknowledged that the GM of the occipital lobes is thicker in ASD than in TD (Pua et al., 2017). For example, both Libero et al. (2015) and Zielinski et al. (2014) report that the **cuneus** has greater cortical thickness in ASD. The latter study also indicates that the **transverse occipital sulci** and **occipital poles** exhibit thicker cortex in ASD. These findings are consistent with our own, which indicate that the surface areas of these structures are larger in ASD compared to TD. The finding according to which ASD patients have greater CD in the transverse occipital sulci is, to our knowledge, new. Because our findings indicate that the surface area of the right cuneus and occipital poles are larger in ASD females than in ASD males—with no such difference being present in TD volunteers—these features may potentially contribute to explaining the interaction between ASD diagnosis and sex. The cunei of both ASD and non-ASD males with highly-repetitive behaviors appear to share structural abnormalities (Focquaert and Vanneste, 2015). Thus, sex-related structural differences in the right cuneus are consistent with previous observations regarding male ASD patients exhibiting more repetitive behavior than female ASD patients (Hartley and Sikora, 2009).

In their review of ASD neuroanatomy, Amaral et al. (2008) lamented the paucity of ASD neuroimaging studies which have adequately-sized samples and proposed that brain imaging studies of ASD should include hundreds of subjects of both sexes. Whereas, a substantial fraction of older ASD neuroimaging studies involve sample sizes as low as ~10 to ~30, the present study is based on MRI and DTI volumes acquired from 193 volunteers. Presumably, this lends much more credence to our findings relative to those of underpowered studies and additionally allows us to more confidently evaluate the merits of SVMs for distinguishing ASD patients from TD subjects based on their neuroanatomic descriptors.

Few previous studies have used ML (in general) and SVMs (in particular) to distinguish between ASD patients and TD volunteers, as well as between individuals of either sex within these populations. Ecker et al. (2010) used an SVM classifier to investigate the predictive value of whole-brain structural volumetric differences between ASD and TD volunteers and obtained a classification accuracy of 81% (compared to ~93% in the present study) based on cross-validation in a sample which was considerably smaller than ours (*N* = 44 vs. *N* = 193 in our case). Compared to our study, Ecker at al. identified a considerably greater number of brain regions which played an important role in distinguishing ASD from TD using SVMs. Furthermore, though our study and theirs agree in identifying medial frontal areas, the inferior frontal gyrus and the parahippocampal gyrus as being important for SVM classification of ASD and TD volunteers, there are discrepancies between our findings and theirs regarding the utility of various neuroanatomic descriptors to the classification process. These and other differences between the two studies could be due to a variety of factors, including (1) heterogeneity across the two samples, (2) differences in the inclusion and/or exclusion criteria, (3) differences between SVM implementations, (4) the sample size difference between the two studies, potentially leading to (5) insufficiently broad sampling of the ASD population in the study with the smaller sample, (6) bias in accurately identifying the neuroanatomic descriptors which are most useful for the purpose of SVM classification, etc. Although the design of an SVM based on the exact parameters adopted by Ecker et al. could provide additional insight into our own results, these two studies' analysis streams and dimensionality reduction techniques differed substantially and likely influenced SVM results appreciably. To provide but one example, we performed dimensionality reduction via PCA whereas Ecker et al. implemented recursive feature elimination with leave-one-out cross-validation. For this reason, it may be misleading to compare the two studies based on SVM design alone; rather, a full replication of the work by Eckert et al. may be necessary to undertake a fair comparison of the two studies. The potential existence of the confounds listed above, however, suggests that further research should be undertaken to understand whether and how ML—and SVMs, in particular—can best be utilized to identify structural brain differences between ASD and TD and between individuals of different sex within these populations.

In conclusion, the present study fills important gaps in the ASD neuroimaging literature and is important because it (1) uses a sample size which is larger than in many previous studies on the topic at hand, (2) suggests useful neuroanatomic measures which are broadly descriptive of ASD-related structural brain abnormalities, (3) accurately identifies such measures whose importance has already been acknowledged by a variety of other studies, (4) uses SVMs to confirm the results of these previous studies which were based on standard statistical approaches, and therefore (5) provides confirmatory evidence to the effect that SVMs are powerful tools for the study of structural abnormalities in ASD.

## Author contributions

AI and JVH: designed the study; AI, XL, CT and ZJ: analyzed the neuroimaging data and implemented statistical analyses; AI, XL, and JVH: interpreted the results of the study; SA: created a computer graphics visualization of the results; AI and XL: wrote the paper with editorial input from JVH, who also secured funding for the study.

### Conflict of interest statement

The authors declare that the research was conducted in the absence of any commercial or financial relationships that could be construed as a potential conflict of interest.
